# Interventions to reduce falls among dialysis patients: a systematic review

**DOI:** 10.1186/s12882-023-03408-7

**Published:** 2023-12-21

**Authors:** Lelise Gute, Edward Zimbudzi

**Affiliations:** 1 Department of Nephrology, Monash Health, VIC, Australia; 2 School of Public Health and Preventive Medicine, Monash University, VIC, Australia; 3 School of Nursing and Midwifery, Faculty of Medicine, Nursing and Health Sciences, Monash University, Level 3, Building 13D, Rm D304, Clayton Campus, 35 Rainforest Walk, Clayton, VIC 3800 Australia

**Keywords:** Falls prevention, Dialysis, Systematic review, Rate of falls, Evidence-based interventions

## Abstract

**Introduction:**

Despite all available evidence regarding increased morbidity and mortality among dialysis patients due to falls and their complications, and an increase in risk factors for falls, relatively little attention has been focused on evidence-based interventions that can reduce falls. We evaluated the effectiveness of fall prevention interventions among dialysis patients.

**Methods:**

We searched Ovid-Medline, Ovid-Embase, PubMed, Cumulated Index to Nursing and Allied Health Literature and the Cochrane Central Register of Controlled Trials (Central) from inception to 19 July 2023 for studies that evaluated the effectiveness of fall prevention interventions among dialysis patients. The search, screening and extraction of data followed standardised processes and the methodological quality of studies was independently assessed by two reviewers. Data was analysed using a narrative synthesis approach.

**Results:**

Of the 18 studies that had full text review, five were eligible. Three studies were performed in the USA and one each in UK and Japan. Four studies were conducted in outpatient hemodialysis centres and one in a hospital-based nephrology unit. Reported sample sizes ranged from 51 to 96 participants per study with a follow-up period of 3 to 35 months. There was moderate-quality evidence that exercises reduce the rate of falls compared to usual care and low to moderate quality of evidence that multifactorial falls prevention interventions reduce the rate of falls. However, treatment effects could not be quantitatively estimated for all interventions due to substantial heterogeneity of included studies.

**Conclusions:**

This systematic review reflects that there is insufficient evidence regarding falls prevention strategies specific to dialysis patients. Available data based on low to moderate quality studies, suggest that among dialysis patients, exercises may reduce falls and the effectiveness of multifactorial interventions such as staff and patient education still need to be explored using high-quality prospective studies.

**Supplementary Information:**

The online version contains supplementary material available at 10.1186/s12882-023-03408-7.

## Introduction

Dialysis patients have a higher risk for falls and of having a serious injury from falls compared to the general population [1–3]. Approximately 25% of dialysis patients fall every year [4] and fall rates of 1.2–1.7 per patient-year among patients treated with hemodialysis (HD) and peritoneal dialysis (PD) respectively have been reported [5]. The high fall rate can be attributed to a combination of aging, kidney disease-related morbidity, HD treatment-related hazards [6] and frailty [7]. While frailty is recognised as one of the primary contributors to the increased risk of falling especially in the older population, among HD patients, there is a strong relationship between frailty and falls regardless of their age [4].

For dialysis patients, falls are associated with an increased risk of fractures due to osteopenia, [8] increased healthcare utilisation [9], institutionalisation, poor health-related quality of life, loss of functional independence, morbidity and mortality [10, 11]. Falls are also associated with increased costs to the healthcare system due to longer lengths of stay by hospitalised dialysis patients, restrictions in mobility and social participation [12, 13].

Despite all available evidence regarding increased morbidity and mortality among dialysis patients due to falls and their complications [11, 12], and an increase in risk factors for falls such as the growing population of older dialysis patients [14, 15], relatively little attention has been focused on evidence-based interventions that can reduce falls. Evaluating the effectiveness of falls prevention strategies that can be specifically used by dialysis patients is important due to several reasons. First, while a number of falls prevention interventions have been used successfully in other populations, dialysis patients have unique risk factors for falls such as micro- and macrovascular complications of diabetes, peripheral vascular disease, cardiovascular disease, high blood pressure with events of low blood pressure related to polypharmacy and fluid shifts during dialysis and vitamin D deficiency [16]. Second, hemodialysis patients often have impaired mobility [17] due to prolonged sitting while having dialysis treatment and some develop postural hypotension and fatigue post treatment [18], which predisposes them to falls. A greater degree of impaired balance among hemodialysis patients is also related to loss of proprioception and peripheral neuropathy [19]. Third, previous research in this population has shown an improvement in some modifiable risk factors such as physical functioning following exercise training [20], but the impact of such interventions on the rate and number of falls among dialysis patients has not been fully ascertained.

To address this research gap, we undertook a systematic review that aimed to evaluate the effectiveness of fall prevention interventions in reducing falls among adult patients on dialysis.

## Methods

The design and conduct of this review and analysis was guided by the Cochrane Handbook for Systematic Reviews of Interventions [21] and the Preferred Reporting Items for Systematic Reviews and Meta-Analyses (PRISMA) guidelines were followed. The protocol of this study was registered on the International Prospective Register of Systematic Reviews (PROSPERO) and is available from https://www.crd.york.ac.uk/prospero/display_record.php, registration number: CRD42023424690. This study involved synthesis of existing data, hence informed consent was not required.

### Search strategy

The Medline, Pubmed, CINAHL, EMBASE databases and all Evidence-Based Medicine (EBM) reviews incorporating the Cochrane Library, Cochrane Database of Systematic Reviews (Cochrane reviews), Database of Abstracts of Reviews of Effects (other reviews), Cochrane Central Register of Controlled Trials (clinical trials), Cochrane Database of Methodology Reviews (methods reviews), the Cochrane Methodology Register (methods studies), Health Technology Assessment Database (technology assessments), NHS Economic Evaluation Database (economic evaluations) and ACP Journal Club were systematically searched from inception to July 19, 2023 for studies meeting the inclusion criteria. We only included papers written in English, due to limited translation resources.

### Selection criteria

The Population, Intervention, Comparison, and Outcome (PICO) framework was used to determine the inclusion and exclusion criteria for this systematic review (Table 1).

**Table 1:** Selection criteria

	**Inclusion**	**Exclusion**
Participants	Adult patients (above 18 years) on dialysis either incentre hemodialysis, home dialysis or peritoneal dialysis)	Patients who are not on dialysis Patients with a kidney transplant
Interventions	Falls prevention interventions that include any of the following; Exercise, rehabilitation, physical therapy, medication management, patient, staff and carer education, environmental reconfiguration Timing: There was no restriction to follow-up duration Intensity: Varying intensity was acceptable Delivery setting: This included clinical areas (dialysis units and wards), primary care and patients’ homes	No intervention or any intervention other than those prespecified in the inclusion criteria
Comparator	Clearly defined usual or standard care with no additional care focusing on falls	
Outcomes	Rate of falls (number of falls per person-years) Number of falls	Lack of at least one relevant prespecified outcome Studies that discusses risk factors
Study design	Randomized controlled trials and systematic reviews of randomized controlled trials, cohort and case–control studies. Published conference abstracts which are described sufficiently with results confirmed as final by authors	Studies that do not report an intervention and studies that report outcome data for one time point Editorials, commentaries, letters and gray literature (e.g., government reports)

### Participants

This review considered studies of adult patients (above 18 years) on either incentre hemodialysis, home dialysis or peritoneal dialysis. In studies where information regarding participants was not clear, we sought clarification from authors. Studies were excluded when this information was not clarified.

### Interventions

For the purpose of this systematic review, falls prevention interventions included any strategies that were utilised to prevent or reduce the rate or number of falls among dialysis patients. Interventions that included exercises, rehabilitation, physical therapy, medication management, patient, staff and carer education and other multifactorial interventions were considered.

### Outcomes

The primary outcomes for this systematic review were the rate of falls (number of falls per person-years) and number of falls. Secondary outcomes included retention of participants in the studies and adherence rates to the interventions and serious adverse events that led to hospitalisation and death. Studies that did not report data regarding the primary outcomes were excluded. Additionally, studies that discussed the effect of falls prevention interventions on risk factors of falls were not considered for this systematic review.

### Search strategy

Three main concepts were used to generate search terms. These were: (i) falls; (ii) adult dialysis patients and (iii) interventions or strategies. For concept 1, the following search terms were used: ‘accidental fall’, fall* or slip* or trip* or collapse*. In concept 2, examples of terms used include ‘exp hemodialysis’, ‘exp peritoneal dialysis’ and ‘exp dialysis’. For concept 3, the following search terms were used: ‘prevention’, ‘intervention’ and ‘strategies’. A detailed search strategy is in Supplementary Table 1.

### Data extraction and critical appraisal

The title and abstract screening as well as full-text reviews were performed by two independent reviewers (LG and EZ). Any disagreements were resolved by mutual consensus. Additionally, reference lists from included texts were reviewed for studies suitable for inclusion. Corresponding authors of the eligible studies were contacted whenever further clarification was required. Data collected included first author’s name, year of publication, country, study design, number of participants, mean age, sex proportion, intervention used, duration of follow-up and the outcomes of interest.

The methodological quality of the included studies was assessed independently by two authors (LG and EZ) using the modified version of the Newcastle–Ottawa scale (NOS) for evaluating the methodological quality of cohort and case–control studies [22] and the Cochrane Risk of Bias tool for assessing the methodological quality of randomised controlled trials [21].

### Data synthesis

A descriptive analysis was performed to summarize data narratively due to clinical and statistical heterogeneity of included studies. Included studies were not sufficiently similar and their quality was variable. There was extensive variation with regards to characteristics of participants, interventions used, reporting of outcome measures and timing of outcome measurement.

## Results

### Study selection

**Fig. 1:**
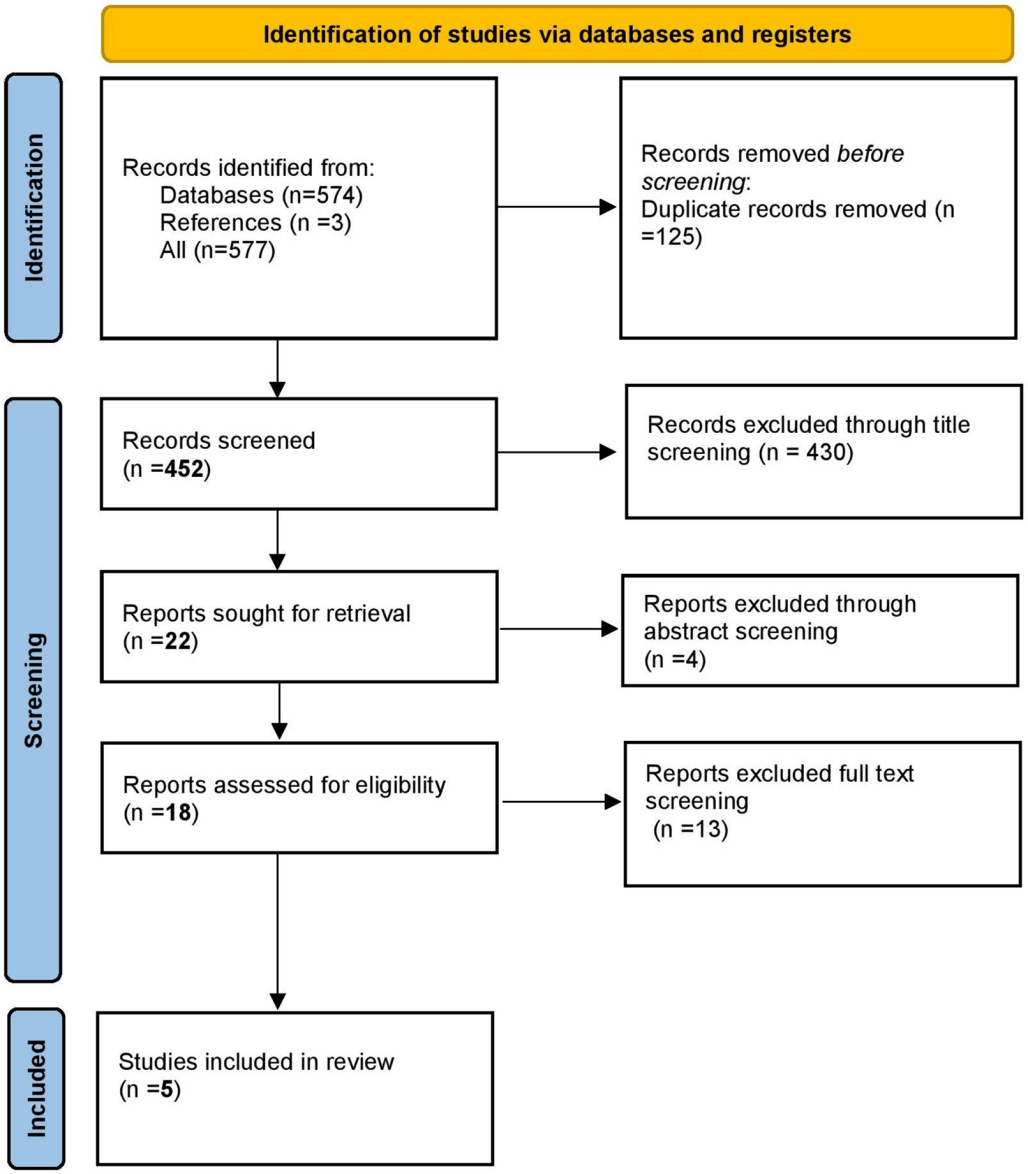
PRISMA flow diagram showing how studies were screened

The results of the search are presented in Fig. 1. Five hundred and seventy-seven citations were identified by the search including three obtained from hand-searching of reference lists. After excluding 125 duplicates, 452 citations were screened for eligibility, from which 430 and 4 were excluded after screening for titles and abstracts respectively. A total of 18 articles were retrieved for full-text review and 13 were excluded because they did not address the intervention. Five studies [23–27] remained and these were included in the systematic review. Two studies [26, 27] without full text but with published conference abstracts were included.

### Study characteristics

Characteristics of the included studies are presented in Table 2. Three studies were performed in USA [23, 24, 26] and one each in UK [25] and Japan [27]. Four studies were conducted in outpatient hemodialysis centres [23–25, 27] and one in a hospital-based nephrology unit [26]. Reported sample sizes ranged from 51 to 96 participants per study with a follow-up period of 3 to 35 months. Three studies [23, 24, 27] utilised patient reports to obtain falls data while one study [25] used medical records and hospital episode statistics and the process was not described clearly in one study [26]. Most of the studies were excluded because they did not specify the intervention or report the number or rate of falls, rather they reported risk factors of falls among dialysis patients (*n* = 17).

**Table 2:** Study characteristics

**Study/Setting**	**N**^**a**^	**Population**	**Intervention** **(Content and delivery characteristics)**	**Control**	**Outcomes and summary of results**	**Follow-up/drop outs/sample size analysed**	**Risk of bias**
Heung et al., 2010 [23] *Cohort study* of Outpatient Hemodialysis Center in USA	N-96 Gender-ND	Adult hemodialysis patients Mean age of those who experienced a fall 62.6 ± 13.4 years, with 7 (58%) being females	Targeted interventions that included the following; 1. Formal staff education program 2. Implementation of fall risk assessment tool 3. Active and passive patient education programs 4. Construction of in-ground scale 5. Full lighting during shift change periods 6. Routine use of towels around dialysis machines to wick leaked fluid 7. Installation of support bars in patient restroom	Period before the interventions (4-year baseline period)	Incidence of falls that occurred at dialysis center’s premises *Results:* There were 2 patient-related falls in the postintervention period, a reduction in fall incidence to 9 (95% CI 0 to 21) falls per 100,000 dialysis treatments (*P* = 0.06)	Follow up: 21 months Drop outs: ND Sample size analysed: ND	Moderate^b^
Malhotra et al., 2023 [24]. *Randomised controlled trial* conducted at single academic outpatient HD facility in the USA	*N* = 55 Male IG-15 CG-13 Female IG-13 CG-14	Participants with ESKD receiving hemodialysis who were able to walk with or without assistive devices	The wearable activity tracker plus structured feedback (intervention) arm. Patients received face-to-face goalsetting counseling in the dialysis facility along with feedback graphs and charts to visualise their progress	Patients had a wearable activity tracker alone (comparator) arm	Step count and absolute change in daily step count, averaged per week, from baseline to completion of 12 weeks intervention *Results*: There were 2 reported falls in the intervention arm and 1 fall in the comparator arm. Falls data was based on patient interviews	Follow up: 3 months Drop outs:9 IG:5 CG:4 Sample size analysed: *n* = 46 IG: *n* = 23, CG: *n* = 23	Moderate^c^
Young et al., 2020 [25] *A mixed-methods* *Randomised controlled trial* conducted at three HD centres within the UK East Midlands Renal Network	*N* = 51 Male: IG-17 CG-22 Female: IG-7 CG-5	Prevalent adult HD patients of the CYCLE-HD trial with a Clinical Frailty Scale Score of 4–7 (vulnerable to severely frail) Mean age 63 ± 12 years	Patients in the intervention group had three times per week supervised, moderate-intensity exercises following a one month run-in. The duration of the intervention was six months. Cycling resistance was progressively increased to maintain the rate of perceived exertion in response to exercise adaptation. Progressive training was also allowed for patients unable to complete 30 min of continuous cycling until the set target was reached	Patients were managed according to the usual centre practice	Primary outcomes were related to feasibility. Secondary outcomes were falls incidence measured from baseline to 1 year following intervention completion, and exercise capacity, physical function, physical activity and patient-reported outcomes measured at baseline and 6 months *Results*: 11 and 5 falls were observed in the CG and IG respectively. The crude falls incident rate ratio (IRR) was 1.95 (95% CI 0.63 to 7.18), suggestive of an almost two-fold increased incidence of falls within the usual care group. Falls data was obtained from medical records and hospital episode statistics	Follow up: 12 months Drop out: *n* = 6 IG: *n* = 4 CG: *n* = 2 Sample size analysed: *n *= 45 IG: *n* = 20 CG: *n* = 25	Moderate^c^
Hiroki et al., 2022 [27]. Single-center, *prospective, non-RCT* (Japan)	*N* = 65 IG-31 CG-34 Gender-ND	HD patients (79.3 ± 6.7 years	The exercise group was offered 3 years of intradialytic exercise training three times a week. The training program involved both resistance and aerobic training exercises. Four types of resistance exercises were performed using an elastic tube. The aerobic exercise program consisted of 20 min of ergometer cycling. The grip strength (GS), leg extremity muscle strength (LES), short physical performance battery (SPPB) score and 10-min walk speed at baseline were evaluated in both groups. These physical functions were re-evaluated each year in the exercise group only	Patients were managed according to the usual centre practice	Physical functioning which was measured at baseline then 1-, 2- and 3-year time points and number of falls observed during follow up *Results*: 8 (9.4%, 1 of exercise, and 7 of control group) falls were observed; one of the cases had a fracture. The exercise group showed no significant differences in any physical functioning between each measurement time point. However, the exercise intervention was significantly associated with a reduction in falls in the Kaplan–Meier survival analysis and log-rank test. Falls data was based on patient interviews at dialysis	Follow up: 35 months (interquartile range: 22–35 months) Drop out: *n* = 12 IG: *n* = 12 CG: *n* = 0 Sample size analysed: *n* = 53 IG: *n* = 19 CG: n = 34	Moderate^b^
Gengler et al., 2020 [26]. Before and after study within a dialysis unit in USA	N-ND Gender-ND	All patients identified as having a high fall risk were placed on the Fall Prevention Bundle. Age-ND	Fall Prevention Bundle This bundle includes evidence-based interventions such as nonskid socks, use of gait belts, bed and chair alarms, hourly rounding, and safety education	Period before the intervention	Rate of falls *Results*: The fall rate went from 4.56 (2018) to 2.98 (2019), a 35% reduction. The falls with injury rate decreased from a rate of 0.99 (2018) to 0.14 (2019), an 86% reduction. It is not clear how falls data was obtained	Follow up: 12 months	High^b^

*RCT* Randomised controlled trial, *IG* Intervention group, *CG* Control group, *HD* Hemodialysis, *ESKD* End stage kidney disease, *ND*No data, *N*^a^ Number of participants, ^b^ risk of bias was assessed by the Newcastle–Ottawa scale, ^c^ risk of bias was assessed by the Cochrane Risk of Bias tool for assessing the methodological quality of randomised controlled trials, * data based on published abstracts

### Interventions

Three studies [24, 25, 27] utilised exercises as an intervention to prevent falls. The first study [24] used a wearable activity tracker plus structured feedback for patients in the intervention arm. Patients received face-to-face goal setting counseling in the dialysis facility along with feedback graphs and charts to visualise their progress. The initial goal setting occurred one week after baseline and this was followed by weekly goal setting process review of date including progression of steps during a dialysis session. This process was facilitated by a healthcare professional that included a nephrologist. Individualised goals were developed and this focused on increasing steps.

The second study [25] utilised intradialytic cycling as an intervention. Patients in the intervention group had three times per week supervised, moderate-intensity exercises following a one month run-in. The duration of the intervention was six months. Cycling resistance was progressively increased to maintain the rate of perceived exertion in response to exercise adaptation. Progressive training was also allowed for patients unable to complete 30 min of continuous cycling until the set target was reached.

The third study [27] followed up participants for three years and intradialytic exercise training occurred three times a week for those in the intervention group while those in the control group were managed according to the usual centre practice. The training program involved both resistance and aerobic training exercises. The aerobic exercise program consisted of 20 min of ergometer cycling. The grip strength, leg extremity muscle strength, short physical performance battery score and 10-min walk speed at baseline were evaluated in both groups. These physical functions were re-evaluated each year in the exercise group only.

One study [23], which is a cohort study of outpatient hemodialysis patients with a follow-up of 21 months reported on staff and patient education and environmental reconfiguration as an intervention to prevent falls. Components of this intervention included a formal staff education program, implementation of falls risk assessment tool, active and passive patient education programs, construction of in-ground weighing scale, adequate lighting during shift change periods, routine use of towels around dialysis machines to wick leaked fluid and installation of support bars in patient restrooms.

Another cohort study utilised a fall prevention bundle as an intervention. This bundle included evidence-based interventions such as non-skid socks, gait belts, bed and chair alarms, as well as hourly rounding, and safety education. These interventions were targeted at patient deemed to be of high falls risk.

### Effect of exercises on falls

Malhotra et.al [24] reported two falls in the intervention arm and one fall in the control group while Yabe and others [27] observed 8 falls (one in the intervention group, and 7 in the control group). The exercise group showed no significant differences in any physical functioning between each measurement time point. However, the exercise intervention was significantly associated with a reduction in falls. Another study [25] reported a crude falls incident rate ratio of 1.95 (95% CI 0.63 to 7.18), suggestive of an almost two-fold increased incidence of falls within the usual care group.

### Effect of multifactorial interventions

Heung and others [23] utilising a comprehensive suite of elements tailored at staff and patient education and environmental reconfiguration reported no significant difference in the incidence of falls for dialysis patients at baseline and post intervention 32 (confidence interval 15 to 49) and 9 (confidence interval 0 to 22) per 100 000 treatments respectively (*P* = 0.06). On the other hand, a falls prevention bundle reported by Gengler and others [26] resulted in a reduction in falls rate from 4.56 to 2.98, which was a 35% decrease in falls rate. The falls with injury rate decreased from a rate of 0.99 to 0.14, an 86% reduction after 12 months.

### Secondary outcomes

#### Retention and adherence

Adherence to interventions was reported by three studies [24, 25, 27] and this ranged from 61.2% to 90%. Reasons for non-adherence included refusal, feeling unwell and pain [25]. Retention rates for the three studies [24, 25, 27] ranged from 81.5% to 88% and some of the reasons for participant drop out were change of treatment modality, ill health, change of dialysis centre and withdrawal of consent [25]. A qualitative exploration of the reasons for withdrawal of consent revealed that participants becoming unwell, longer duration of the trial and the research not meeting participants expectations were major concerns. Participants suggested establishing rapport and effective communication with the research team might help retention of participants in future trials [25].

#### Serious adverse events

One study [25] whose intervention was based on three times per week supervised, moderate-intensity intradialytic cycling evaluated serious adverse events (SAE) that occurred during the study period. In total, n = 13 (25%) had a SAE, n = 8 (33%) in the exercise group and n = 5 (19%) in the usual care group. Reasons for these SAEs included vascular access complications (*n* = 3, 17%), stroke (*n* = 3, 17%), acute coronary syndrome (*n* = 2, 11%) and non-specific chest pain (*n* = 2, 11%). All SAEs resolved following hospitalisation and there was no death reported. Additionally, none of the SAEs were directly related to the intervention or trial. In another study [24] that utilised a wearable activity tracker plus structured feedback in the intervention arm, four participants were hospitalised (2 each in the intervention and control group) and two deaths were reported (1 each in the intervention and control group). Among the eight patients who had a fall from a study by Yabe and others [27], one sustained a fracture.

#### Risk of bias in included studies

Table 2 presents an overview of the risk of bias for studies assessed using the Cochrane Risk of Bias tool [24, 25] and a modified version of NOS for three cohort studies [23, 26, 27]. The risk of bias ranged from moderate to high. The main issues identified for the two randomised controlled trials were performance and detection bias. Participants and research personnel and outcome assessors had knowledge of the allocated interventions. For the three cohort studies, there was limited evidence that the analysis was controlled for confounders.

## Discussion

In this systematic review of five studies among dialysis patients, there was moderate-quality evidence that exercises reduce the rate of falls compared to usual care. The types of exercises that were effective included intradialytic cycling and wearing of an activity tracker plus structured feedback to encourage individualised goal setting. There was low to moderate quality of evidence that multifactorial falls prevention interventions reduce the rate of falls. These interventions included staff and patient education, environmental reconfiguration, use of non-skid socks, gait belts, bed and chair alarms and hourly rounding. However, treatment effects could not be quantitatively estimated for all interventions due to substantial heterogeneity of included studies.

Our findings suggest that exercises may reduce the rate of falls among dialysis patients. This is consistent with results from other studies among older people living in the community [28–30], older adults receiving geriatrician-led care [31] and healthy older adults [32]. However, the components of the exercises that are effective for particular groups of patients need to be ascertained. Evidence suggests that exercises delivered as a unimodal intervention, particularly resistance training, are effective [33], but the long term effects of such interventions is variable with some studies reporting effects that last for up to two years after an exercise intervention [34]. Another challenge is that exercise interventions are often associated with lower rates of participation with dropout rates of 20%-50% within the first 3–6 months [35, 36] having been reported and individuals who dropout are likely to be those most in need of regular exercise. To improve participation, exercise interventions that are frequent, of moderate duration, accessible and convenient, socially acceptable and individually tailored [37] should be prioritised.

While interventions that included staff and patient education and environmental reconfiguration did not improve the rate of falls among dialysis patients, this result may not have been statistically significant due to lack of power. Additionally, it is known that falls occur mostly in unfamiliar environments [38] and in this regard dialysis units may be protective against falls given that dialysis patients are in these setting more frequently. Nevertheless, there is limited evidence to indicate where falls among adult dialysis patients most commonly occur. Another study that reported on the effectiveness of multifactorial falls prevention interventions reported a significant improvement in falls incident rate ratio after 12 months. Multifactorial falls prevention interventions can potentially improve the rate of falls if delivered appropriately. To do this, well-designed educational programs for health professionals on falls prevention in institutional settings need to be well-documented [39] and the content, delivery mode and educational design principles and models that are effective need to be investigated. Whilst patient education is an aspect of most hospital falls prevention programs, few studies have evaluated the outcomes or design of educational components, based on educational theory [40] even though benefits of delivering hospital patient education informed by educational theory and the principles of health behaviour change are known [41].

These findings need to be considered in light of the low to moderate quality of evidence examined. Reasons include marked heterogeneity of included studies with regards to study size, duration and intensity of interventions and variability related to the assessment of the outcomes. Reporting bias may also have occurred especially in three studies that used patient reports as a source of falls data. There was also potential bias in the methodological conduct of the studies because we included studies of any design as well as published abstracts (with no full texts) to capture most interventions that are being used to prevent falls among dialysis patients. Additionally, for HD patients, frailty is an important contributor to severe outcomes such as fractures and poor quality of life [42] making the evaluation of the independent effect of falls prevention interventions on the rate of falls difficult. However, a study that included patients with a wide spectrum of comorbidities and advanced age, including frail patients who otherwise would have been excluded from other exercise trials showed that a combined endurance and resistance intradialytic exercise training over 12 months improved physical function, reduced hospital days, and was feasible and safe [43].

The review has a number of strengths. To our knowledge, this is the first systematic review to evaluate the effectiveness of falls prevention interventions among dialysis patients. Additionally, the conduct of this this review is underpinned by methodological rigor and the use of reliable tools. The approach we used minimised publication bias as we attempted to retrieve all available literature on this subject by not limiting inclusion to study design. We also contacted study authors to ensure that accurate data were included. Limitations include exclusion of studies published in other languages other than English.

Our findings have several implications to practice and research. First, we have shown that there is a paucity of research that evaluates the effectiveness of interventions to prevent falls among dialysis patients. Well-designed randomised controlled trials and prospective longitudinal studies that assess the rate and number of falls as primary outcomes are required to address this gap in research. These studies should also examine where falls among dialysis patients are likely to occur as well as exploring falls interventions in home, hospital and community environments. Second, new interventions that are tailored to individual capabilities need to be considered. Interventions, that embrace the use of technology may also be effective in certain subgroups of the dialysis population, however, these need to be evaluated.

In conclusion, this systematic review reflects that there is insufficient evidence base regarding falls prevention strategies specific to dialysis patients even though they have a higher risk of falls compared to other population groups. However, available data based on low to moderate quality studies, suggest that among dialysis patients, exercises may reduce falls and the effectiveness of multifactorial interventions such as staff and patient education still need to be explored using high-quality prospective studies.

### Supplementary Information

**Additional file 1:** **Table S1.** Search terms.

## Data Availability

All data generated or analysed during this study are included in this published article [and its supplementary information files].

## Abbreviations

HD: Hemodialysis
PD: Peritoneal dialysis
PRISMA: Preferred reporting items for systematic reviews and meta-analyses guidelines
PROSPERO: International prospective register of systematic reviews
CINAHL: Cumulative index of nursing and allied health literature
EMBASE: Excerpta Medica database
EBM: Evidence-based medicine
ACP: American College of Physicians journal Club
NHS: National Health Service
