# Supplementary material for: Interventions to reduce falls among dialysis patients: a systematic review
Source: BMC Nephrol. 2023 Dec 21;24:382. doi: 10.1186/s12882-023-03408-7 (PMC10734056; doi:10.1186/s12882-023-03408-7)
Supplement: Supplementary file 1 — Additional file 1: Table S1. Search terms. [file 12882_2023_3408_MOESM1_ESM.docx]

**Supplementary Table 1: Search terms**

|  | **Search terms** |
| --- | --- |
| 1 | exp Dialysis/ or exp Peritoneal Dialysis/ or exp Peritoneal Dialysis, Continuous Ambulatory/ or exp Renal Dialysis |
| 2 | exp Renal Replacement Therapy/ |
| 3 | exp Kidney Failure, Chronic/ |
| 4 | exp Renal Insufficiency/ |
| 5 | or/1-4 |
| 6 | exp "Physical and Rehabilitation Medicine"/ or exp Rehabilitation/ or exp Rehabilitation Nursing/ or exp Rehabilitation Research/ or exp Hospitals, Rehabilitation/ |
| 7 | exp "Patient Education as Topic"/ |
| 8 | staff education.mp. |
| 9 | exp Medication Therapy Management/ |
| 10 | exp Exercise/ or exp Exercise Therapy/ |
| 11 | (falls prevention and control).mp. |
| 12 | falls prevention intervention*.mp. |
| 13 | or/6-12 |
| 14 | exp Accidental Falls/ |
| 15 | (Fall and Slip).mp. |
| 17 | Falls.mp. |
| 18 | or/14-17 |
| 19 | 5 and 13 and 18 |
